# Supplementary material for: Effects of Hypoxemia by Acute High-Altitude Exposure on Human Intestinal Flora and Metabolism
Source: Microorganisms. 2023 Sep 11;11(9):2284. doi: 10.3390/microorganisms11092284 (PMC10535934; doi:10.3390/microorganisms11092284)
Supplement: Supplementary file 1 [file microorganisms-11-02284-s001.zip › Figure S1.pdf]

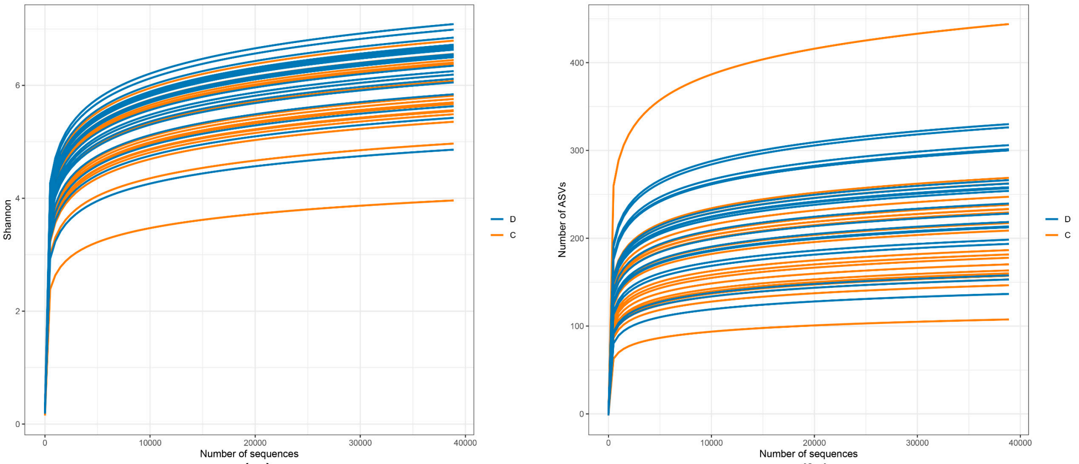

(a) (b)

Figure S1. AVS analysis. (**a**) Shannon–Wiener curves of the C and D groups; (**b**) Number of AVSs in the C and D groups.
